# Measuring Dynamics of Infant-Adult Synchrony Through Mocap

**DOI:** 10.3389/fpsyg.2019.02839

**Published:** 2019-12-18

**Authors:** Zamara Cuadros, Esteban Hurtado, Carlos Cornejo

**Affiliations:** Laboratorio de Lenguaje Interacción y Fenomenología, Escuela de Psicología, Pontificia Universidad Católica de Chile, Santiago, Chile

**Keywords:** interpersonal synchrony, mocap, anatomical imitation, mirroring, dyadic interaction

## Abstract

The temporal dynamics of parent-infant synchrony have been well documented. In recent years, the introduction of more accurate technologies for tracking movements has allowed the distinction of different morphological patterns of dyadic coordination. However, the potential of these technologies to explore infant-adult synchrony has not yet been explored. In the present study, we examined the temporal, spatial, and morphological synchrony patterns of infant-unknown adult pairs participating in a storytime session by a motion capture system (mocap). We find low but significant correlation levels of body synchrony between infants and unknown adults. This synchronized coactivity adopted two differentiated forms: mirror-like and anatomical. While the infants’ movements mirrored those of the adults with a lag (0.9 s), the adults’ reactions to the infants were anatomical with delay (0.4 s). This evidence could contribute novel insights to rethink synchrony and its measurement.

## Introduction

The spontaneous tendency to synchronize body movements in time, space, and form seems to be a ubiquitous feature in face-to-face social encounters (Bernieri and Rosenthal, 1991; Rio and Warren, 2016; Cornejo et al., 2017a). It has been observed in social exchanges between known and unknown adults (Latif et al., 2014; Cornejo et al., 2018), musical improvisations between unknown preschoolers (Endedijk et al., 2015), and daily life routines between parents and their infants (Reyna et al., 2012). In the field of child development, infant-adult harmonious synchronization at the sub-second time scale was initially documented by Condon and Sander (1974). They conducted a microanalysis to examine video recordings of infant-caretaker interactions. Independent judges segmented and coded the films into time frames according to the changes in movement direction identified for each interactant. The codification of synchrony attended to “process units” of movement, such as inclination, rotation, and flexion of all body parts that are perceived in movement. Then, correspondences between the changes in the movement direction of both interactants were analyzed frame by frame. After verifying interjudges’ reliability, the researchers reported that neonates synchronized their bodily movements with adult speech as early as the first day of life.

Since that pioneering study, a growing body of research has attempted to capture mother-infant or father-infant synchrony in social settings through microanalysis. Contemporary studies have included some variations in the procedure stated by Condon and Sander. For example, software and behavioral microcoding guides have been introduced to code, score, and compute different magnitudes of the synchrony temporal dimension. Therefore, the quantification of synchrony has increased in efficiency and objectivity. In addition to estimating concurrences between dyadic events by means of descriptive measures (e.g., frequencies, durations, and latencies), studies conducted in this field have calculated synchrony from probabilistic measures (e.g., conditional probability) and time series (e.g., degree of coherence and the direction of influence between the two corresponding time series).

Studies exclusively using descriptive measures of synchrony often compare the matching frequency or duration of different parent-infant behaviors among groups, e.g., the matching of facial expressions between mother-twins vs. mother-singletons (Kokkinaki and Markodimitraki, 2019) and father-infant vs. mother-infant (Kokkinaki and Vasdekis, 2015). Probabilistic measures have been incorporated to study the brain basis of synchrony (Atzil et al., 2014), the role of parental oxytocin in infant-parent coordination (Gordon et al., 2010), and the effects of mother-infant synchrony on infant physiological and behavioral regulation (Pratt et al., 2015). Time series measurement and analysis have been used in studies examining the concordance between infant-parent biological and behavioral rhythms (Feldman et al., 2011), as well as the consequences of early mother-infant synchronization for the development of self-control (Feldman et al., 1999), symbolic play and internal state talk (Feldman and Greenbaum, 1997), and empathy (Feldman, 2007). They have also been implemented to inquire how mother-infant coordination is impacted by their physiological states (Busuito et al., 2019), maternal hair cortisol levels (Tarullo et al., 2017), and emotional dysregulation in mothers with mood disorders (Lotzin et al., 2016).

An alternative technique to capture infant-parent interactions is behavioral coding. This method differs in coding procedures from microanalysis. Trained raters codify and score synchrony by means of global or specific scales (Delaherche et al., 2012; Leclere et al., 2014). Behavioral coding studies often analyze synchrony according to descriptive measures calculated from the raw data of the scale. By means of behavioral coding, synchrony has been studied as a mediator of relations between attachment and mind-related comments (Lundy, 2003) and attachment and infant cardiac vagal tone (Waters and Mendes, 2016). Behavioral coding has been utilized to study factors that modulate mother-infant behavioral synchrony, such as maternal anxiety (Moore et al., 2016), maternal nurturance and child emotional negativity (Skuban et al., 2006). Finally, behavioral coding has been used to codify and describe mother-preterm infant synchrony during feeding (Reyna et al., 2012).

Together, findings from microanalytical and behavioral coding studies have provided a better understanding of dyadic synchrony sensitivity to parental physiological and psychological factors. In particular, this research has gathered valuable knowledge about the temporal dynamics of coordinated behaviors between mother-infant and father-infant interactions. Studies analyzing time series have made it possible to identify the simultaneity of dyadic synchronizations, as well as the time lags of infant behaviors to those of his/her mother, and vice versa. Importantly, they have achieved to establish that the correlations at time-delay between infant-mother and infant-father behaviors range from 0.16 to 0.20 (Feldman, 2003, 2007; Feldman et al., 2011). This finding suggests that infant-caregiver interactions display higher correlations than those informed between familiar and unfamiliar adults chatting – ranging from 0.01 to 0.11 (Boker et al., 2002; Ramseyer and Tschacher, 2011; Paxton and Dale, 2013a; Cornejo et al., 2018). In sum, the temporal dynamics of parent-infant synchrony are well documented.

By contrast, we still do not know much about the morphological dynamics of interpersonal synchrony between children and adults. This is important since information on the morphology of coordination can reveal aspects on its nature and function (Bernieri and Rosenthal, 1991; Semin and Cacioppo, 2008). An example is the finding of mirror-like and anatomical coordination, two types of time-delayed forms of coordination originally identified in studies on goal-directed imitation (Gleissner et al., 2000; Chiavarino, 2012). Mirror-like coordination involves movements that reflect those of the model, establishing a spatial correspondence between them (Chiavarino, 2012). For instance, the imitator moves her left hand when the model moves her right hand. Conversely, anatomical coordination comprises movements that reconstruct the model body-scheme, establishing an anatomical equivalence between them (Chiavarino, 2012). For example, the imitator moves her right hand while the model moves her right hand. In spontaneous interactions, these morphological patterns of coordination have only been described in pairs of known and unknown adults engaging in conversations (Cornejo et al., 2018). Importantly, it has been suggested that anatomical imitation – and not the mirror-like one – may be related to perspective taking skills (Dunphy-Lelii, 2014; Pierpaoli et al., 2014). Furthermore, infants diagnosed with autist spectrum disorder performing low in emotional understanding show mirror-like imitation impaired, but not the anatomical one (Avikainen et al., 2003).

On the other hand, the examination of synchronization between infants and unknown adults has received little attention. Bernieri et al. (1988) conducted one of the few studies addressing this relationship. Consequently, two questions remain unanswered: (1) Do coordinative dynamics emerge in face-to-face interactions between an infant and an unknown adult? If this is the case, (2) Which form adopts the simultaneous and delayed synchronization between the adult and infant? Considering the higher accuracy of some current techniques for capturing synchrony, we explored both questions by analyzing the coordination patterns between an unknown adult and 14-month-old infants, who are just beginning to increase motor independence. We designed a study to address two objectives: (1) To inquire if, by using cutting-edge technology, we can capture synchrony between infants and unknown adults, and (2) To describe the temporal, spatial and morphological patterns of such synchronization during a storytime session. Given the limited utility of observational methods for the accurate measurement of the forms of synchronization, we used a motion capture system (henceforth: mocap) to track dyadic interactions. Through this technique, a previous study with adults succeeded in recording accurate and detailed measurements of the dyadic synchrony attributes (Cornejo et al., 2018), such as time (e.g., zero-lag or time-delayed), space (e.g., amplitude and direction of synchronized motions), and form (e.g., mirror-like and anatomical coordination).

As we have reviewed above, research shows that interpersonal synchrony seems to be a ubiquitous phenomenon in social exchanges between people regardless of their familiarity level. Considering this, in the present study we expected to trace its dynamics into an unexplored type of relationship: Infant-unknown adult.

## Materials and Methods

### Participants

Twenty-two 14-month-old infants (12 girls; *M* age = 14.2 months; *SD* = 0.2 months) were recruited from Family Health Centers in Santiago de Chile, and day nurseries at the Chilean National Board of Kindergartens and the Pontificia Universidad Católica de Chile. We select 14-month-old infants for two reasons. First, at that age, infants already have some motor independence. Second, numerous literature reports indicate that at the beginning of the second year of life, infants cooperate with strangers and help them reach their goals (Warneken and Tomasello, 2006, 2007; Warneken et al., 2007; Fawcett and Liszkowski, 2012; Cirelli et al., 2014a, b, 2016, 2017; Tunçgenç et al., 2015; Fawcett and Tunçgenç, 2017; Cirelli, 2018). All infants included in the study were typically developing and were able to walk unassisted. Infants recruited had parents without any diagnosis of mental disorders. From the original sample, three infants were excluded from the final analyses because disengaged from the interaction or became distressed. The study was approved by the Ethical Committee of Social Sciences at the Pontificia Universidad Católica de Chile and Ethical Committee of Metropolitan Health Service South-East. Informed consent was obtained from all parents. The infants took part in a storytime session with one of the two unknown female adults (*M* age = 21.5 years old; *SD* = 0.7 years old).

### Apparatus

**FIGURE 1:**
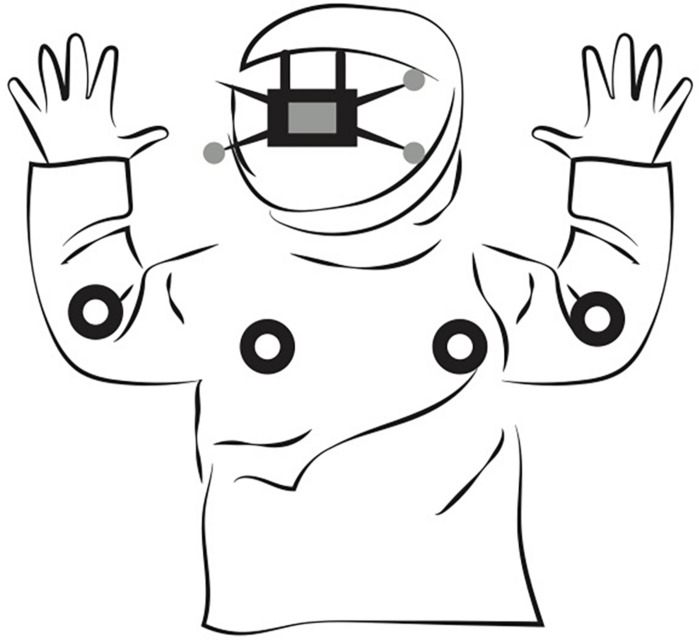
Illustration of the markers located on infants’ bodies. The infants used a comfortable sweater and hat that had the reflective markers attached to the key positions: the upper back (2×), the elbows (2×), and the head (3×).

Body movements were recorded with a mocap consisting of 36 NaturalPoint Prime-41 purpose-specific cameras and a personal computer running Motive software supplied by the manufacturer of the cameras. The cameras were located close to the ceiling, surrounding the rectangular perimeter of a room (width 3 × depth 4 × height 2 m.). The mocap fills a room with infrared light and tracks the position of small infrared reflective spherical markers. To increase participants’ comfort, we followed the recording protocol by Cornejo et al. (2018). Seven reflective markers were positioned on each person’s body: the upper back (2×), the elbows (2×), and the head (3×). We used an elastic band to hold the small reflective markers around the unknown adult’s body. For the infants, we used a comfortable sweater and hat that had the markers attached to the key positions (see Figure 1).

### Picture Storybook

Bérengère Delaporte’s short story “Where is Mom Elephant?” was shown and told by the unknown adult to the infants. The story narrates a baby elephant who searches for his mother in the forest until he finds her.

### Procedure

**FIGURE 2:**
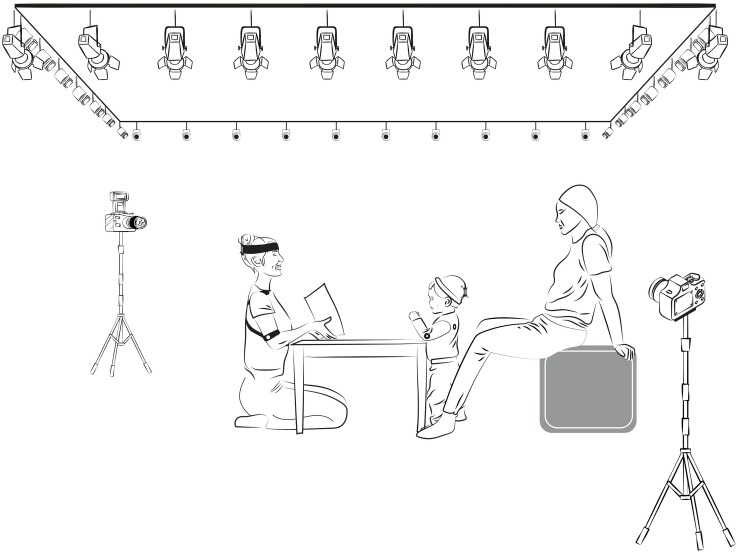
Depiction of the room setting and spatial disposition of the participants. 36 Natural Point Prime-41 purpose-specific cameras were located close to the ceiling, forming a rectangular perimeter above and surrounding the participants. During the reading session, the infant and the unknown adult were located in front of each other around a square table separating them. The parents were sitting behind the infants, with their hands resting on the chair to avoid covering the reflective markers, and their feet supported on the table legs to surround them.

Parents and infants were invited to the laboratory at a time when the infant was expected to be rested and fed. Upon arrival, an assistant familiarized them with optical and video cameras in the room She described and demonstrated the mocap operation to parents before explained the study procedure. Parents received detailed instructions about their role and position during the sessions. Parents were told that: (1) during the familiarization, they could play freely with infants, and (2) during the storytime, they should prevent disrupting the activity. Parents were asked to remain silent and sitting behind the infants – as shown in Figure 2 – to avoid covering the reflecting markers, touch or move the infant’s body. They were also asked to help keep their infant attentive to the story the unknown adult is telling (e.g., by pointing to the storyteller and saying “Look,” whenever the infant turns toward them or became distracted).

After being instructed, parents were asked (1) to sit behind infants, who were standing up around a square table, and (2) to encourage them to play with the toys placed on the table. The unknown adult was seated on one side of the table and already had the reflective markers positioned on her body (see Figure 2). The unknown adult was instructed to exhibit a gentle face and attitude and to play in front of the infant. She could not interact directly with him/her. While the infant played, the assistant asked the parents to dress the infant in the sweater and hat. She told him/her that the story session was about to begin and reminded the instructions. After that, the assistant removed the toys from the table and handed a book to the unknown adult. The unknown adult began the storytelling. She was instructed to show the book characters to the infant and to encourage him/her to be involved during the reading session by performing expressions, voices, and sounds of the story characters. The book was read in its entirety in approximately 145 s on average (the duration range was from 126 to 151 s). Parents did not disrupt the reading session, touch or move the infant’s body. After the storytelling, parents were asked to rate, on a 1–7 scale, the level of engagement perceived in the infant. The parental reports indicate high infant linking toward the reading session (*M* = 6.25, *SD* = 0.85), and the unknown adult (*M* = 6.42, *SD* = 0.88).

### Preprocessing

The first 120 s of each interaction was selected for preprocessing. We exported data from the Motive software. Then, we used custom scripts to trajectorize the markers. The mocap data for each couple were manually labeled by corresponding body parts and identified the participant to which each marker belonged. Finally, we visually inspected the results.

### Computation of Speed Cross-Correlation Curves

Our data consisted of a collection of position time series pairs; each pair consisted of one time series for the positions of the adult and another one for the positions of the infant. First, we averaged the two back markers for each participant into a single 3D position and only kept 1D positions in the proximity axis (the direction that lies between the two subjects). Second, we computed discrete speed signals (distance over time within each measurement period) by taking a marker’s 1D position at each frame and subtracting its position from the previous frame. Third, a single-pole 10-Hz cutoff frequency low-pass filter was applied to the speed signals to remove fast recording artifacts without affecting typical human motion which focuses on a slower spectral range (Zeng and Zhao, 2011), since typical human motion spectral content is restricted to less than 10 Hz in frequency (Zeng and Zhao, 2011). Fourth, for each pair of time series, we computed a normalized cross-correlation curve (i.e., in a Pearson correlation scale from −1 to 1) as follows:

$$r\,\left[\Delta \,t\right]=\sum_{t=-\infty }^{\infty }a\,\left[t-\Delta \,t\right]\,b\,\left[t\right]$$

Here, *r* is correlation, Δ*t* is the time lag (horizontal axis of the cross-correlation curve), and *a*[*t*] is a time series that contains centered and standardized speeds of the averaged two markers of the back for subject A, indexed by discrete time *t*, where values before the beginning and after the end of the recording are zero. Similarly, *b*[*t*] contains the same information for subject B. Correlations were computed only for time offsets between −1.5 s and +1.5 s. Finally, all cross-correlation curves were aggregated into a single pooled curve as a weighted average. For each correlated pair of time series, correlation *r*[Δ*t*] was weighted by the inverse of standard deviations:

$$\frac{1}{\sqrt{\sum_{t=-\infty }^{\infty }\left(I\,\left[t\right]\,A\,{\left[t-\Delta \,t\right]}^{2}\right)}\,\sqrt{\sum_{t=-\infty }^{\infty }\left(I\,\left[t\right]\,B\,{\left[t\right]}^{2}\right)}}$$

Here, *A*[*t*] and *B*[*t*] subjects A and B speed time series, respectively, which are centered but not standardized, and are zero-valued outside the recording period. The value of *I* is 1 where *A*[*t*−Δ*t*]*B*[*t*] is non-zero, and 0 otherwise (in order to only pool by values that were actually used to compute *r*[Δ*t*]). This approach to aggregating correlation curves has several advantages to simple averaging. It normalizes against different motion intensities between subject couples and it excludes the effect of any differences in average speed (which are expected to be small but could be non-zero). Most importantly, the result is also a correlation (instead of a simple average of correlations), so it can be statistically tested with usual techniques.

### Statistical Inference for Cross-Correlation Values

Pearson correlation values in cross-correlation curves were tested for differences with respect to zero. A confidence interval was plotted around the cross-correlation curve. The correlations were statistically significant if their confidence intervals did not touch the zero-correlation horizontal line. We used the Fisher transform for *p*-values and confidence interval computation as detailed in Cornejo et al. (2018), based on a Holm–Bonferroni correction for the 31 correlation values in a cross-correlation curve, to control the family wise error rate. The alpha level was set at 0.001.

### Interpretation of Aggregated Cross-Correlation Curves

In the plot of the resulting cross-correlation curves, the vertical axis corresponds to the Pearson correlation, while the horizontal axis corresponds to the time delays. Therefore, a relatively high magnitude correlation at *t* = 0 is consistent with a tendency for couples to display an immediate coordination pattern (i.e., they move in a similar fashion and at the same time). When the same occurs at a positive time, it can be interpreted as a delayed coordination, where the infant tends to display a motion pattern similar to that of the adults but at a later time. Conversely, notable correlations at negative times are associated with the adults imitating the infants [see Cornejo et al. (2018) for examples and more details]. The axes have been arranged so that the positive correlation values indicate mirror-like coordination between the adult and infant. Conversely, negative correlation values correspond to anatomical coordination between the adult and infant.

## Results

**FIGURE 3:**
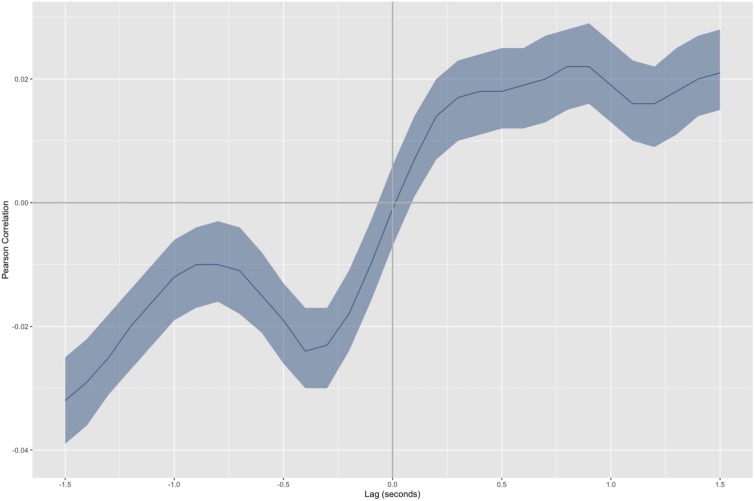
Aggregated cross-correlation curve for back markers average in the proximity axis. The colored area surrounding the curve indicates the confidence interval. Data have been organized so that positive correlation values indicate to mirror-like coordination between the interactants. Conversely, negative correlation values correspond to anatomical coordination between interactants. The positive lag times in the plots correspond to the infant’s reactions to the unknown adult. Conversely, negative lag times correspond to the adult’s reactions to the infant.

Figure 3 displays a cross-correlation curve for the motion data captured during the tale reading session. Two correlation peaks are evident in the curve. The first peak is at *t* = -0.4 s, *r* = -0.024 (*p* < 0.001). This peak indicates that the adult tends to imitate the infant with a 0.4 s lag. Since the correlation value is negative, anatomical coordination is evidenced. Thus, the adult’s movements reconstruct the infant body scheme with a delay (e.g., when the infant moves to the left, the adult moves to the left 0.4 s later). The second peak occurs at *t* = 0.9 s, *r* = 0.022 (*p* < 0.001). It corresponds to the infant’s reactions to the adult with a 0.9 s lag. Since the correlation value is positive, the coordination pattern evidenced is mirror-like in this case. Thus, the infant’s movements reflect the adult’s actions with a lag (e.g., when the adult moves to the left, the infant moves to the right 0.9 s later).

There were no statistically significant effects of the infant’s age [on anatomical synchrony: *F*_(__1_,_19__)_ = 0.021, *p* = 0.883; on mirror-like: *F*_(__1_,_19__)_ = 0.507, *p* = 0.485] and the unknown interacting adult [on anatomical synchrony: *F*_(__1_,_19__)_ = 0.111, *p* = 0.743; on mirror-like synchrony: *F*_(__1_,_19__)_ = 0.033, *p* = 0.857].

## Discussion

The current study measured the synchrony of infant-unknown adult pairs by mocap and investigated the patterns of their synchronized coactivity during a storytime session. Although previous research has studied the temporal-spatial attributes of mother-infant synchrony by observational methods (e.g., microanalysis and behavioral coding), mocap technology additionally allows exploring the morphology of synchrony with high accuracy (Cornejo et al., 2017b). Using this device, we captured and analyzed measures of synchrony such as latency (i.e., simultaneous or delayed), direction (i.e., whether the infant’s movements follow those of the adult’s and vice versa) and form (i.e., mirror-like or anatomical). As a result, we were able to provide evidence that could contribute novel insights to rethink synchrony and its measurement.

An important finding of our study is that infants entered a synchronized dynamic in their face-to-face encounters with an unknown adult. This result is in line with the results found by Bernieri et al. (1988). They reported synchrony between mothers and unknown babies during spontaneous interactions. However, they also found that this synchrony was weaker than that between mothers and their sons. The same result was obtained by Feldman et al. (2011) when comparing the synchrony between mother-infant dyads and pseudosynchrony created from random mother-infant dyads. However, our results do not allow us to know if the synchrony found is similar to previously reported parent-infant synchrony. Future research will need to determine concordances or differences between parent-infant synchrony and infant-stranger synchrony.

Additionally, our result differs from those of Bernieri et al. (1988) in both the conceptualization and the measurement of synchrony. Since they used a behavioral coding system, synchrony was interpreted as the coincidence of discrete behavioral states between interactants. In contrast, the method to capture the interactions in the present study (mocap) allowed us to examine the synchrony in terms of the degree to which the body movements are coordinated in time, space, and form (Bernieri and Rosenthal, 1991). Hence, our research extends the previous work insofar as it characterizes in detail interactions between infant and unfamiliar adult pairs. We observed four characteristics of dyadic synchrony: (1) the infant-adult movement correlations were low in magnitude, although statistically significant; (2) the synchronized coactivity occurs with lags in both directions, i.e., infant-leads–adult-follows and adult-leads–infant-follows; (3) the form adopted by the delayed coordination of infants toward the adult is mirror-like; and (4) the shape of the delayed coordination of the adult concerning infants is anatomical.

First, the magnitude of the correlations between the infant-adult movements that we found is lower than those reported by studies using microanalysis. For example, there have been informed correlations between infant-mother and infant-father behaviors from 0.16 to 0.20 (Feldman, 2003, 2007; Feldman et al., 2011). Presumably, these differences are derived from the capture method used but not from the type of relationship observed. When using a mocap, more reliable temporal-spatial measurements are obtained from movements, including those that are invisible to the human eye. Thus, it is likely that the highest accuracy in the capture method improved the estimation of correlations (Cornejo et al., 2017b). The magnitudes we observed are in fact consistent with earlier reports of interpersonal synchrony between adults measured by means of accurate techniques. In particular, similar low but significant correlation levels have already been reported in studies on spontaneous coordination between friends and strangers interacting in social contexts (Boker et al., 2002; Ramseyer and Tschacher, 2011; Cornejo et al., 2018). However, more research is needed to substantiate our presumption. Future research could benefit from comparing the results of dyadic coordination obtained through micro-analytic coding and mocap methods.

Our results add evidence in favor of the ubiquity of synchrony in face-to-face social interactions. Synchrony has been noted in diverse social settings (Valdesolo et al., 2010; Paxton and Dale, 2013a; Rodrigues and Passos, 2013; Tschacher et al., 2014) and among people who are and are not acquainted with each other (Latif et al., 2014; Cornejo et al., 2018). Considering that the correlation magnitudes observed here are similar to those reported in adult studies, our findings lead to questions concerning the evolution of synchrony through the lifespan. Future longitudinal studies could offer a revealing perspective on the evolution of synchrony over time and within different kinds of social relationships.

Second, while the bidirectionality described is not novel in itself, it should be noted that it is the first time that the direction of synchrony is calculated based on an accurate and reliable measurement of the behaviors of the interactants. Previous research has identified that the synchrony direction from microanalytic coding has achieved a valuable contribution to the existing field of studies. However, it must also face the inherent difficulties of microanalysis, such as it is time-consuming and labor-intensive, and it critically depends on the interjudges’ reliability of the codification performed (Delaherche et al., 2012; Paxton and Dale, 2013b; Leclere et al., 2014). It is possible that mocap is not the least expensive alternative, but unlike microanalysis and behavioral coding, it favors automation and accuracy in capturing interaction data.

The third and fourth characteristics of dyadic synchrony are concerned with two types of time-delayed body coordination: mirror-like and anatomical. These modalities of body synchrony have been previously described in the literature about goal-directed imitation (Chiavarino, 2012; Dunphy-Lelii, 2014; Pierpaoli et al., 2014) and, more recently, by a study of spontaneous interactions between pairs of conversing adults (Cornejo et al., 2018). In our results, both types are found in spontaneous interactions between adults and infants. The mirror-like delayed synchrony of infants and the anatomical synchrony of adults seem to replicate a trend previously reported by the field of imitation. Several studies show that, in the absence of instructions about how to imitate, children, adolescents, and adults tend to imitate the movements of the model in a mirror-like way (Wapner and Cirillo, 1968; Bekkering et al., 2000; Barchiesi and Cattaneo, 2013; Dunphy-Lelii, 2014; Ubaldi et al., 2015). However, adults make fewer mistakes when asked to imitate the model anatomically (Wapner and Cirillo, 1968; Chiavarino, 2012; Pierpaoli et al., 2014). Our results do not allow us to establish direct comparisons between the forms adopted by the spontaneous infant-adult synchrony and the mirror-like and anatomical imitations from studies that instruct the reproduction of a model’s movements. Nevertheless, they inaugurate a promising avenue to explore the relationships between synchrony and imitation, as well as their impact on child development.

Incidentally, the study of interpersonal synchrony between infants and unfamiliar adults opens an encouraging scenario to explore and discuss evolutionary hypotheses on the foundations of cooperation with social peers and the roots of childcare. If interpersonal synchrony is ubiquitous to face-to-face encounters between people, regardless of their degree of familiarity, is it a consequence of humans’ increasing social interdependence? (Warneken and Tomasello, 2009a, b; Tomasello et al., 2012). Does coordination arise from a breeding system in which the members of a social group (individuals other than parents) help to care for offspring? (Hrdy, 2008, 2009; Burkart, 2009; Burkart et al., 2009). The answers to these questions exceed the scope of this article, but we point out the convenience of interpersonal synchrony study as a promising way to discuss them.

In summary, the measurement of synchrony using a mocap not only provided the possibility of accurately measuring the phenomenon but also favored the knowledge of attributes declared in the synchrony definitions not fully explored by previous works. Future research could benefit from the use of novel techniques currently available to record interactions. As a case in point, it could prove the utility of intermediate alternatives between observational and mocap methods, such as automated video analysis or magnetic motion capture systems.

## Data Availability Statement

The full datasets used for this article are not publicly available because of restrictions imposed by the informed consent assigned by participants. Requests to access the datasets should be directed to CC, cca@uc.cl.

## Ethics Statement

All infants’ parents gave written informed consent in accordance with the Declaration of Helsinki. The protocol was approved by the Ethical Committee of Social Sciences at the Pontificia Universidad Católica de Chile and Ethical Committee of Metropolitan Health Service South-East from Santiago de Chile.

## Author Contributions

ZC, EH, and CC made contributions to the conception of the manuscript, data collection, data analysis, participated in the writing process by adding substantively relevant content, approved the final version to be published, and agreed to be accountable for all aspects of the text in ensuring that questions related to the accuracy or integrity of any part of the work are appropriately investigated and resolved.

## Conflict of Interest

The authors declare that the research was conducted in the absence of any commercial or financial relationships that could be construed as a potential conflict of interest.
